# Anti-Inflammatory Components of the Starfish *Astropecten polyacanthus*

**DOI:** 10.3390/md11082917

**Published:** 2013-08-13

**Authors:** Nguyen Phuong Thao, Nguyen Xuan Cuong, Bui Thi Thuy Luyen, Tran Hong Quang, Tran Thi Hong Hanh, Sohyun Kim, Young-Sang Koh, Nguyen Hoai Nam, Phan Van Kiem, Chau Van Minh, Young Ho Kim

**Affiliations:** 1 Institute of Marine Biochemistry, Vietnam Academy of Science and Technology (VAST), 18 Hoang Quoc Viet, Nghiado, Caugiay, Hanoi 10000, Vietnam; E-Mails: thaonp@imbc.vast.vn (N.P.T.); cuongnx@imbc.vast.vn (N.X.C.); cuongnx@imbc.vast.vn (B.T.T.L.); cuongnx@imbc.vast.vn (T.H.Q.); cuongnx@imbc.vast.vn (T.T.H.H.); cuongnx@imbc.vast.vn (N.H.N.); cuongnx@imbc.vast.vn (P.V.K.); cuongnx@imbc.vast.vn (C.V.M.); 2 College of Pharmacy, Chungnam National University, Daejeon 305-764, Korea; 3 School of Medicine, Brain Korea 21 Program, and Institute of Medical Science, Jeju National University, Jeju 690-756, Korea; E-Mails: just_so@naver.com (S.K.); yskoh7@jejunu.ac.kr (Y.-S.K.)

**Keywords:** starfish, *Astropecten polyacanthus*, IL-12 p40, IL-6, TNF-α, LPS-stimulated BMDCs

## Abstract

Inflammation is important in biomedical research, because it plays a key role in inflammatory diseases including rheumatoid arthritis and other forms of arthritis, diabetes, heart disease, irritable bowel syndrome, Alzheimer’s disease, Parkinson’s disease, allergies, asthma, and even cancer. In the present study, we describe the inhibitory effect of crude extracts and steroids isolated from the starfish *Astropecten polyacanthus* on pro-inflammatory cytokine (Interleukin-12 (IL-12) p40, interleukin-6 (IL-6), and tumor necrosis factor α (TNF-α)) production in lipopolysaccharide (LPS)-stimulated bone marrow-derived dendritic cells (BMDCs). Among those tested, compounds **5** and **7** showed potent inhibitory effects on the production of all three pro-inflammatory cytokines with IC_50_ values ranging from 1.82 ± 0.11 to 7.00 ± 0.16 μM. Potent inhibitory activities were also observed for compound **1** on the production of IL-12 p40 and IL-6 with values of 3.96 ± 0.12 and 4.07 ± 0.13 μM, respectively, and for compounds **3** and **4** on the production of IL-12 p40 with values of 6.55 ± 0.18 and 5.06 ± 0.16 μM, respectively. Moreover, compounds **2** (IC_50_ = 34.86 ± 0.31 μM) and **6** (IC_50_ = 79.05 ± 2.05 μM) exhibited moderate inhibitory effects on the production of IL-12 p40, whereas compounds **3** (IC_50_ = 22.80 ± 0.21 μM) and **4** (IC_50_ = 16.73 ± 0.25 μM) moderately inhibited the production of TNF-α and IL-6, respectively.

## 1. Introduction

Inflammation is a complex set of interactions among soluble factors and cells that can occur in any tissue in response to traumatic, infectious, post-ischemic, toxic, or autoimmune injury. Inflammation is defined as part of a complex biological response of vascular tissue toward exogenous harmful stimuli [1] and is mediated by a variety of soluble factors, including a group of secreted polypeptides known as cytokines, which play a key role in the modulation of immune responses. 

Interleukin-12 (IL-12) is a pro-inflammatory cytokine produced by activated antigen-presenting cells, dendritic cells, monocytes/macrophages and B cells in response to bacterial products and immune signals [2]. Originally identified as a B-cell differentiation factor, interleukin-6 (IL-6) is now known to be a multifunctional cytokine that participates in several biological events, including immune responses, hematopoiesis and acute-phase reactions [3]. Some of the regulatory effects of IL-6 involve the inhibition of tumor necrosis factor (TNF) production, providing negative feedback and limiting the acute inflammatory response [4,5]. Cytokines such as IL-6 are essential but their constitutive overproduction is often involved in various diseases, which accounts for the negative regulatory mechanism in the IL-6 signaling system [6,7].

TNF has since been implicated in diverse inflammatory, infectious and malignant conditions, and the importance of TNF in inflammation was demonstrated by the efficacy of anti-TNF antibodies or administration of soluble TNF receptors (TNFRs) in controlling rheumatoid arthritis and other inflammatory conditions [8,9]. TNF is not typically detectable in healthy individuals, but elevated serum and tissue levels are observed under inflammatory and infectious conditions [10]. Also, serum levels correlate with the severity of infections [9]. Therefore, inhibiting the expression and production of powerful mediators, including IL-12 p40, IL-6, and TNF-α by anti-inflammatory components could represent a preventive or therapeutic target, and may be used to develop anti-inflammatory agents for health promotion and disease prevention.

Starfish are found in all oceans. There are over 1500 known species, and many remain undiscovered. Forcipulatida, Paxillosida, Platyasterida, Spinulosida, and Valvatida are the main subclasses of Asteroidea. Starfish have been investigated by organic chemists, biochemists, and pharmacologists as a potential source of bioactive marine natural products. Various secondary metabolites including steroids, steroidal glycosides, anthraquinones, alkaloids, phospholipids, peptides, and fatty acids have been reported from starfish [11]. They are also a major source of marine products widely distributed in all oceans, and yield a large number of unique bioactive metabolites such as steroids and saponins. These compounds with unique structures are known to possess anti-tumor, anti-inflammatory [12], immunomodulation, anti-allergy, anti-fungal, hemolytic [13], antimutagenic [14], neuritogenic [15], cytotoxic, and anti-viral [16] activities.

**Figure 1:**
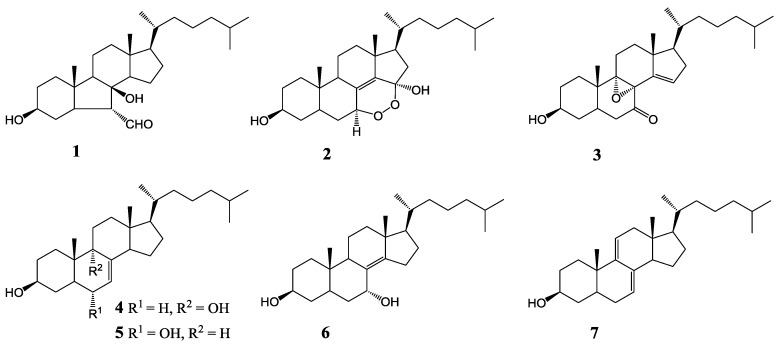
Structures of steroids **1** to **7** from starfish *Astropecten polyacanthus*.

The starfish *Astropecten polyacanthus* is an invertebrate in the order spinulosa, class Asteroidea, and phylum Echinodermata. *Astropecten* species are abundant in the Vietnamese sea, and have been used as tonic agents in Vietnamese folk medicine. In previous studies, we isolated and identified seven steroids (**1**–**7**) from *A. polyacanthus* (Figure 1), some of which had cytotoxic effects against HL-60 (leukemia), PC-3 (prostate), and SNU-C5 (colorectal) human cancer cells [17]. In continuation of our investigations on Vietnamese marine organisms regarding anti-inflammatory activity [18,19,20,21], we found that the MeOH extract and CH_2_Cl_2_ partition showed significant *in vitro* anti-inflammatory activities. The present study deals with the inhibitory capacity of steroids isolated from the starfish *A. polyacanthus* on LPS-induced expression of the pro-inflammatory cytokines IL-12 p40, IL-6, and TNF-α in bone marrow-derived dendritic cells.

## 2. Results and Discussion

Bone-marrow derived dendritic cells (BMDCs) play a key role in the interface between the innate and acquired immune systems [22]. Activated BMDCs perform crucial functions in immune and inflammatory responses via the pathogen-associated molecular patterns (PAMPs)-stimulated production of pro-inflammatory cytokines such as IL-12 p40, IL-6, and TNF-α.

Among its many biological activities, IL-12 provides an obligatory signal for the differentiation of effector T-helper 1 (Th1) cells and the secretion of Th1 cytokines, gamma interferon (IFN-γ) and IL-2. IL-12 plays an important role in the generation of a Th1 response against human pathogens [23,24]. Although the induction of IL-12 by intracellular organisms is necessary for a protective host Th1 response, overexpression of Th1 cytokines and IL-12 may contribute to the development and perpetuation of chronic inflammatory and autoimmune diseases. Thus, understanding the regulated expression of IL-12 in macrophages may provide insight into the pathogenesis of infectious and inflammatory diseases, and could reveal novel approaches to alter immune responses [25].

**Figure 2:**
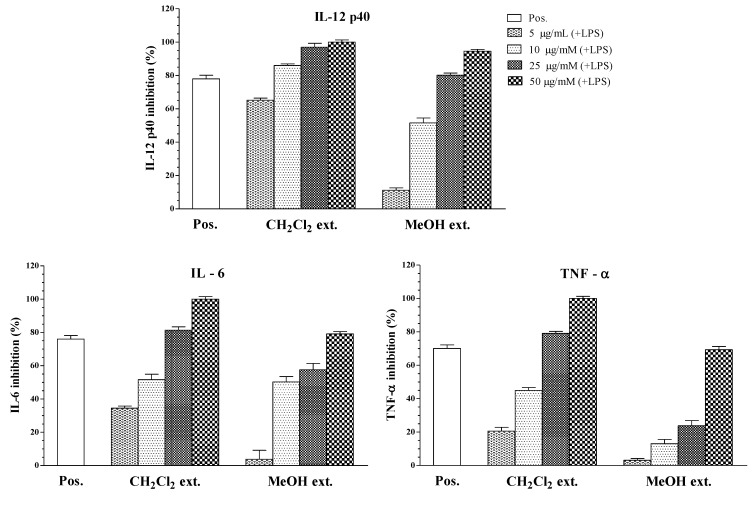
Effect of crude extracts (5, 10, 25, 50 μg/mL) on IL-12 p40, IL-6, and TNF-α production by LPS-stimulated BMDCs. The data were presented as inhibition rate (%) compared to the value of vehicle-treated DCs.

In this study, the MeOH extract significantly inhibited the production of all pro-inflammatory cytokines, while the dichloromethane fraction showed more potent inhibitory effects (Table 1 and Figure 2). The methanol extract from the starfish *A. polyacanthus* showed inhibitory activity on IL-12 p40, IL-6, and TNF-α production (IC_50_ = 11.47 ± 0.16, 20.28 ± 0.22, and 36.99 ± 0.24 μg/mL, respectively). Since the methanol extract had inhibitory activity on IL-12 p40 production, it was partitioned in dichloromethane/water to obtain a dichloromethane soluble portion and an aqueous phase. Based on Table 1, the dichloromethane soluble extract showed potent inhibitory activity towards LPS-stimulated IL-12 p40 production (IC_50_ = 1.27 ± 0.11 μg/mL), which was higher than that by the methanol extract (IC_50_ = 11.47 ± 0.16 μg/mL). 

**Table 1:** Anti-inflammatory effects of the extracts on LPS-stimulated BMDCs.

Fraction Extracts	IC_50_ Values (μg/mL) ^a^		
	IL-12 p40	IL-6	TNF-α
CH_2_Cl_2_ extract	1.27 ± 0.11	8.82 ± 0.18	11.48 ± 0.16
Crude MeOH extract	11.47 ± 0.16	20.28 ± 0.22	36.99 ± 0.24
**SB203580** ^b^	2.52 ± 0.12	1.67 ± 0.13	3.65 ± 0.12

^a^ IC_50_ values for selected compounds are given in column IL-12 p40, IL-6, and TNF-α; ^b^ Positive control; values <100 μg/mL are considered to be active.

**Figure 3:**
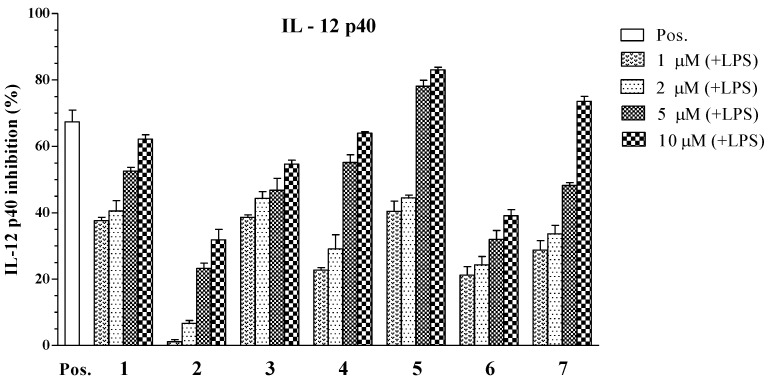
Effect of steroids **1** to **7** (1, 2, 5, 10 μM) on IL-12 p40 production by LPS-stimulated BMDCs. The data were presented as inhibition rate (%) compared to the value of vehicle-treated DCs.

Subsequently, all isolated compounds (**1**–**7**) from the CH_2_Cl_2_ fraction of *A. polyacanthus* were tested for inhibitory effects on the production of the pro-inflammatory cytokines IL-12 p40, IL-6, and TNF-α. Of those tested, compounds **1**, **3**–**5**, and **7** showed potent inhibition on IL-12 p40 production with IC_50_ values of 3.96 ± 0.12, 6.55 ± 0.18, 5.06 ± 0.16, 1.82 ± 0.11, and 3.90 ± 0.14 μM, respectively (Table 2). This variability in inflammatory response inhibition by **1**, **3**–**5**, and **7** may be explained by the different levels of secreted inflammatory factors upon LPS stimulation. Among those tested, compound **5** (a steroid with hydroxyl group at C-6) had the greatest inhibitory activity towards LPS-stimulated IL-12 p40 production (IC_50_ = 1.82 ± 0.11 μM), which was comparable to that of the positive control, SB203580 (IC_50_ = 5.00 ± 0.16 μM). Compound **5** is an isomer of **4**, but **5** showed potent inhibitory activity at the tested concentrations. Compounds **2** and **6** showed moderate suppressive effects on the production of IL-12 p40 by 34.86 ± 1.31, 79.05 ± 2.05 μM respectively (Table 2 and Figure 3), relative to the vehicle group. These compounds were subjected to evaluation of their effects at various concentrations on the production of the pro-inflammatory cytokines in LPS-stimulated BMDCs.

**Table 2:** Anti-inflammatory effects of compounds **1** to **7** on LPS-stimulated BMDCs.

Compounds	IC_50_ Values (μM) ^a^		
	IL-12 p40	IL-6	TNF-α
**1**	3.96 ± 0.12	4.07 ± 0.13	>100
**2**	34.86 ± 1.31	>100	>100
**3**	6.55 ± 0.18	>100	22.80 ± 0.21
**4**	5.06 ± 0.16	16.73 ± 0.25	>100
**5**	1.82 ± 0.11	5.76 ± 0.14	4.94 ± 0.12
**6**	79.05 ± 2.05	>100	>100
**7**	3.90 ± 0.14	2.61 ± 0.10	7.00 ± 0.16
**SB203580 ^b^**	5.00 ± 0.16	3.50 ± 0.12	7.20 ± 0.13

^a^ IC_50_ values for selected compounds are given in column IL-12 p40, IL-6, and TNF-α; ^b^ Positive control; values <100 μM are considered to be active.

**Figure 4:**
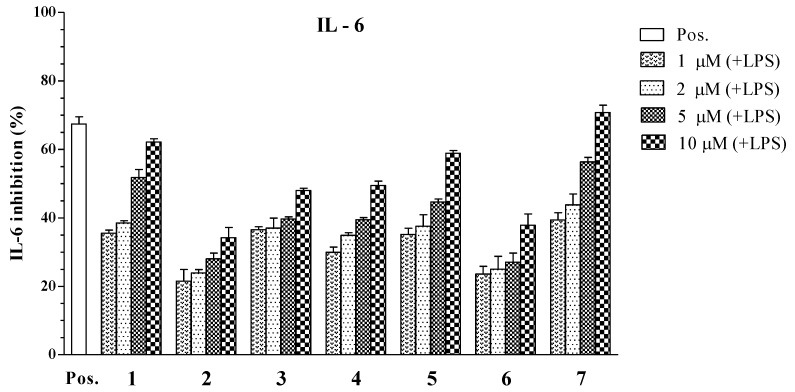
Effect of steroids **1** to **7** (1, 2, 5, 10 μM) on IL-6 production by LPS-stimulated BMDCs. The data were presented as inhibition rate (%) compared to the value of vehicle-treated DCs.

As a result, the dichloromethane-soluble extract showed inhibitory cilliary neurotropic factor activity of LPS-stimulated IL-6 production (IC_50_ = 8.82 ± 0.18 μg/mL). Compounds **1**, **4**, **5**, and **7** considerably decreased the production of IL-6 in the LPS-stimulated BMDCs with IC_50_ values ranging from 2.61 ± 0.10 to 16.73 ± 0.25 μM (Table 2). Remarkably, compound **7** significantly inhibited IL-6 production with IC_50_ values of 2.61 ± 0.10 μM. The inhibitory effects of compounds **1** and **5** were similar to the positive control (IC_50_ = 3.50 ± 0.12 μM), with IC_50_ values of 4.07 ± 0.13 and 5.76 ± 0.14 μM, respectively. Compound **4** showed moderate inhibition of IL-6 production, with IC_50_ values of 16.73 ± 0.25 μM. The remaining compounds did not show significant activity (IC_50_ > 100 μM) against IL-6 production (Figure 4).

**Figure 5:**
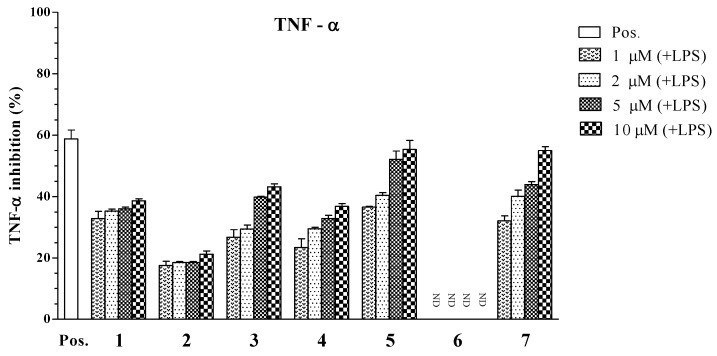
Effect of steroids **1** to **7** (1, 2, 5, 10 μM) on TNF-α production by LPS-stimulated BMDCs. The data were presented as inhibition rate (%) compared to the value of vehicle-treated DCs.

Overexpression of pro-inflammatory cytokines TNF-α and IL-6 is associated with the development of autoimmune, inflammatory, and immunopathological diseases. Therefore, blocking TNF-α, IL-6, and their respective signaling pathways can be effective for treatment of inflammatory diseases. The TNF-α assay results are shown in Table 2. Compounds **5** and **7** showed potent inhibitory effects on the production of TNF-α, with IC_50_ values of 4.94 ± 0.12 and 7.00 ± 0.16 μM, respectively. The inhibitory effects of compound **3** (IC_50_ = 22.80 ± 0.21 μM) was moderate compared to the positive control (IC_50_ = 7.20 ± 0.13 μM), while compounds **1**, **2**, **4**, and **6** did not show significant inhibitory effects on TNF-α production (IC_50_ > 100 μM, Figure 5).

## 3. Experimental Section

### 3.1. Biological

The sample of starfish *A. polyacanthus* was collected at Cat Ba, Haiphong, Vietnam, in June 2012 and identified by Do Cong Thung (Institute of Marine Environment and Resources, VAST, Hanoi, Vietnam). A voucher specimen (AsP-06-2012_01) was deposited at the Institute of Marine Environment and Resources and Institute of Marine Biochemistry, VAST.

### 3.2. Cell Cultures and Measurement of Cytokine Production

In this study, we used LPS-stimulated BMDCs as a model for testing the inhibitory effects of fractions and isolated compounds on the secretion of pro-inflammatory cytokines IL-12 p40, IL-6, and TNF-α. BMDCs (1 × 10^5^) were seeded in 48-well plates at 37 °C, 5% CO_2_ for 1 h, and then treated for 1 h with the compounds at concentrations of 1, 2, 5, and 10 μM, followed by stimulation with LPS (10 ng/mL, Figure 2). The supernatants were harvested 16 h after stimulation and IL-12 p40 secretion was measured using enzyme-linked immunosorbent assay (ELISA). SB203580, an inhibitor of cytokine suppressive binding protein/p38 kinase, was used as a positive control [26]. SB203580 inhibited IL-12 p40, IL-6, and TNF-α production with IC_50_ values of 5.00 ± 0.16, 3.50 ± 0.12, and 7.20 ± 0.13 μM, respectively.

Bone marrow-derived dendritic cells were grown from wild-type C57BL/6 mice (Orient Bio Inc., Seoul, Korea) as previously described [27]. All animal procedures were approved by and performed according to the guidelines of the Institutional Animal Care and Use Committee of Jeju National University (#2010-0028). Briefly, the mouse tibia and femur was obtained by flushing with Dulbecco’s modified Eagle medium to yield bone marrow cells. The cells were cultured in Rosell Park Memorial Institute (RPMI) 640 medium containing 10% heat-inactivated fetal bovine serum (FBS; Gibco, Grand Island, NY, USA), 50 μM β-mercaptoethanol, and 2 mM glutamine supplemented with 3% J558L hybridoma cell culture supernatant containing granulocyte-macrophage colony-stimulating factor (GM-CSF). The culture medium was replaced with fresh medium every other day. At day six of culture, non-adherent cells and loosely adherent dendritic cell (DC) aggregates were harvested, washed, and resuspended in RPMI 1640 supplemented with 5% FBS. The BMDCs were incubated in 48-well plates in 0.5 mL containing 1 × 10^5^ cells per well, and then treated with the isolated compounds at different concentrations for 1 h before stimulation with 10 ng/mL LPS from *Salmonella minnesota* (Alexis, Famingdale, NY, USA). Supernatants were harvested 18 h after stimulation. Concentrations of murine TNF-α, IL-6, and IL-12 p40 in the culture supernatant were determined by ELISA (BD PharMingen, San Diego, CA, USA) according to the manufacturer’s instructions.

IL-12 p40 level in unstimulated DC: not detectable. IL-12 p40 level in LPS-stimulated DC: 51.34 ± 0.66 (ng/mL). IL-6 level in unstimulated DC: not detectable. IL-6 level in LPS-stimulated DC: 41.12 ± 2.38 (ng/mL). TNF-α level in unstimulated DC: not detectable. TNF-α level in LPS-stimulated DC: 1.83 ± 0.02 (ng/mL). 

The inhibitory activity (*I*) was expressed as the inhibition rate (%), which was calculated from the following formula:

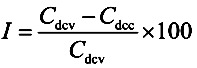



*C*_dcv_: Cytokine level (ng/mL) in vehicle treated DC; *C*_dcc_: Cytokine level (ng/mL) in compound treated DC.

The data are presented as means ± standard deviation (SD) of at least three independent experiments performed in triplicate.

## 4. Conclusions

In conclusion, compounds **5** and **7** showed potent inhibitory effects on the production of all three pro-inflammatory cytokines, with IC_50_ values ranging from 1.82 ± 0.11 to 7.00 ± 0.16 μM. Potent inhibitory activities were also observed for compound **1** on the production of IL-12 p40 and IL-6, and for compounds **3** and **4** on the production of IL-12 p40. Moreover, **2** and **6** exhibited moderate inhibitory effects on IL-12 p40 production, whereas **3** and **4** moderately inhibited TNF-α and IL-6 production respectively. The structures of **1**–**7** suggested that the hydroxy group at C-6, the conjugated double bond structures, and the presence of an aldehyde group in the steroidal skeleton might play an important role in the anti-inflammatory effects of these compounds. Further studies are required to assess the mechanisms of action and their potential use as new anti-inflammatory agents. These results support the use of starfish steroid components to inhibit pro-inflammatory cytokine secretion, including IL-12 p40, IL-6, and TNF-α, and to prevent and treat inflammatory diseases.
